# RNA-sequencing Reveals Global Transcriptomic Changes in *Nicotiana tabacum* Responding to Topping and Treatment of Axillary-shoot Control Chemicals

**DOI:** 10.1038/srep18148

**Published:** 2015-12-16

**Authors:** Sanjay K. Singh, Yongmei Wu, Jayadri S. Ghosh, Sitakanta Pattanaik, Colin Fisher, Ying Wang, Darlene Lawson, Ling Yuan

**Affiliations:** 1 Kentucky Tobacco Research and Development Center , University of Kentucky, Lexington, KY 40546, U.S.A; 2 Department of Plant and Soil Sciences, University of Kentucky, Lexington, KY 40546, U.S.A.; 3 South China Botanical Garden, Chinese Academy of Sciences, Guangzhou, China; 4 R J Reynolds, Inc. 950 Reynolds Blvd, Winston-Salem, NC 27102, U.S.A.

## Abstract

Removal of terminal buds (topping) and control of the formation of axillary shoots (suckers) are common agronomic practices that significantly impact the yield and quality of various crop plants. Application of chemicals (suckercides) to plants following topping is an effective method for sucker control. However, our current knowledge of the influence of topping, and subsequent suckercide applications, to gene expression is limited. We analyzed the differential gene expression using RNA-sequencing in tobacco (*Nicotiana tabacum*) that are topped, or treated after topping by two different suckercides, the contact-localized-systemic, *Flupro*^*®*^ (FP), and contact, *Off-Shoot-T*^*®*^. Among the differentially expressed genes (DEGs), 179 were identified as common to all three conditions. DEGs, largely related to wounding, phytohormone metabolism and secondary metabolite biosynthesis, exhibited significant upregulation following topping, and downregulation after suckercide treatments. DEGs related to photosynthetic processes were repressed following topping and suckercide treatments. Moreover, topping and FP-treatment affect the expression of auxin and cytokinin signaling pathway genes that are possibly involved in axillary shoot formation. Our results provide insights into the global change of plant gene expression in response to topping and suckercide treatments. The regulatory elements of topping-inducible genes are potentially useful for the development of a chemical-free sucker control system.

Many plant species produce a single stalk with an apical shoot bud that grows predominantly, and suppresses the growth of the axillary shoots, a phenomenon known as apical dominance. Decapitation or removal of the apical bud (a process known as topping) is an important agronomic practice that impacts the quality and yield of a number of crop species. Crop plants including cotton (*Gossypium* spp.)^1^, sunn hemp (*Crotolaria juncea*)^2^, fenugreek (*Trigonella foenum-graecum*)^3^, mustard (*Brassica* spp.)^4^ and okra (*Abelmoschus esculentus*)^5^, benefit from this agronomic practice. Recent studies suggest topping reduces bollworm infestation, and increases cotton boll number and lint yield^6^. However, topping also stimulates axillary shoot proliferation that competes with the primary shoot for water and nutrients thus potentially diminishing yields. Studies indicate that axillary shoots negatively affect the photosynthetic efficiency and fruit set in tomato^7^,^8^. Like many other crops, tobacco (*Nicotiana tabacum*) exhibits a strong apical dominance. Topping and control of axillary shoot (sucker) proliferation are common agronomic practices in tobacco^9^,^10^. Topping improves the physical and chemical characteristics of the cured leaves. Suckers reduce quality and interfere with mechanical harvesting of tobacco leaves^9^,^10^. Control of axillary shoot formation is, therefore, crucial to maximize the beneficial effects of topping.

Control of sucker formation can be achieved by hand topping or chemical treatment. Hand topping is time-consuming, labor-intensive, and expensive. Application of chemicals is thus the most common method for sucker control. Currently, three types of commercial sucker control chemicals (suckercides) are regularly used: contacts (*e.g. Off-shoot-T*^*®*^, OS), systemics (*e.g. Maleic hydrazide*, MH), and contact-localized-systemics (*e.g. Flupro*^*®*^, FP). Long chain fatty alcohols are the principal ingredients of contact suckercides. They break down the plasma membrane, resulting in dehydration and death of the suckers^11^. Systemic suckercides are absorbed by the leaves and translocated to the leaf axils where they inhibit cell division and axillary shoot development. Flumetralin is one of several dinitroaniline suckercides that have contact-localized-systemic action. It is absorbed when it comes in contact with plant tissues and hinders the growth of axillary buds by inhibiting cell division. Chemical suckercides are used on different crops, including tomato^7^,^8^, bougainvillea^12^, red pepper^13^, and tobacco^14^,^15^,^16^, to control axillary shoot growth and to maximize yield.

Being sessile, plants are constantly challenged by various abiotic and biotic factors, and have thus evolved complex mechanisms to combat and survive under adverse conditions. Wounding of plants, caused by abiotic (*e.g.* mechanical damage/topping) or biotic (*e.g.* insect and herbivore) factors, triggers significant changes in the gene expression and metabolism as part of the healing and/or defense process. The phytohormone, jasmonic acid (JA), is known as a key regulator of wound signaling in plants. In addition, a number of compounds, including oligopeptides, oligosaccharides, and reactive oxygen species, act in the wound signaling pathway to protect plants from the adverse conditions^17^,^18^. The phytohormone, auxin, has long been known as a major signal molecule in apical dominance. Auxin is synthesized in the shoot apex especially in young leaves and transported to base by a polar auxin transport system^19^. Topping affects the polar auxin transport and stimulates the proliferation of dormant axillary shoot buds in the leaf axils. Cytokinin promotes axillary shoot growth, possibly acting as a secondary messenger for auxin^20^. Although topping and application of sucker control chemicals are common agronomic practices for some crops, their influences on expression of genes related to different metabolic and developmental processes have not been thoroughly investigated. There are only a few reports on the influence of topping on gene expression in tobacco. A previous study identified 129 topping responsive genes in roots of flue-cured tobacco using suppression subtractive hybridization (SSH), and found that the majority of the differentially expressed genes (DEGs) are related to secondary metabolism, stress/defense, signal transduction, and protein metabolism^21^. Small non-coding RNAs, including microRNAs (miRNAs) and siRNAs, regulate numerous biological processes in plants. Recent studies suggest that expression of miRNAs are also affected following topping^21^,^22^,^23^. Sequencing of small RNA libraries from tobacco roots has identified several topping responsive miRNAs. The targets of the differentially expressed small RNAs, are predicted to regulate a number of developmental processes, including leaf and lateral shoot initiation, root development, floral organogenesis and flowering time, seed development, microtubule organization, and cytokinesis. In addition, the small RNAs also play roles in plant’s response to stress, hormones, nitrogen and carbon metabolism, signal transduction, and nucleic acid metabolism^22^,^23^. The effects of suckercides on gene expression remain unknown.

In this study, we applied RNA-seq and analyzed DEGs in tobacco plants that were either topped or treated with one of two suckercides, OS and FP, after topping. This study provides insights into global changes in gene expression in response to topping and suckercide application, and advances our understanding of how chemicals inhibit axillary shoot formation. The knowledge gained will aid our ongoing effort to develop chemical-free axillary shoot control systems.

## Materials and Methods

### Tissues and RNA isolation

The burley tobacco *(Nicotiana tabacum* variety TN 90 LC) plants were grown at 22 °C under a 16/8-h photo-period in an environmentally controlled greenhouse. The contact suckercide, *Off-Shoot-T*^®^, and contact-localized-systemic suckercide, *Flupro*^®^, were purchased from Chemtura Agro Solutions, USA. OS and FP were applied as 5% (v/v) and 2% (v/v) solutions in water respectively, to eight week-old tobacco plants. For topping, the flowering head and two leaves below the flag leaf were removed when the first flower of inflorescence was about to open^24^. FP or OS was applied to individual plants immediately after topping^25^. The leaf samples were collected after 24 h from the control (un-topped), topped and suckercide-treated plants, frozen immediately in liquid nitrogen and stored at −80 °C until RNA extraction (Fig. S1).

Total RNA was isolated from 100 mg of leaf tissues using the RNeasy Plant Mini Kit (Qiagen, Chatsworth, USA) following manufacturer’s instructions. RNA quantity was determined using a NanoDrop ND-1000 spectrophotometer (NanoDrop Technologies, Wilmington, DE, USA). Quality of the RNA samples was determined using Agilent 2100 Bioanalyzer (Agilent Technologies, Palo Alto, CA, USA) and RNA samples with RNA integrity number (RIN) above 8 were used for library preparation.

### Illumina sequencing and data processing

Two microgram of RNA from each sample (n = 2/sample) was sent to the Sequencing and Genotyping Center at the Delaware Biotechnology Institute in the University of Delaware for RNA-Seq library preparation and sequencing. The cDNA libraries were prepared using the TruSeq RNA Sample Prep Kit (Illumina, San Diego, CA) according to the manufacturer’s protocol. The libraries were then pooled together and sequenced on an Illumina HiSeq 2500. Deep sequencing was performed on duplicates for each treatment (total eight samples) for a 50 cycle single end run. The data quality was checked at the Sequencing and Genotyping Center at the Delaware Biotechnology Institute at the University of Delaware and sequencing reads were provided as in FASTq format.

Raw Illumina sequence reads were processed using the prinseq-lite-0.20.4^26^ for removal of low-quality reads. Finally, preprocessed reads were assessed for quality control with FastQC (version 0.11.3; Babraham Bioinformatics, Cambridge, UK).

### Data analysis

Reads were mapped to a reference tobacco genome available from Sol Genomics Network^27^ using CLC Genomics Workbench v. 5.5.1 (CLC bio, Aarhus N, Denmark) following the manufacturer’s instructions. At least 95% of the bases were required to align to the reference and a maximum of two mismatches were allowed. DEGs were identified using the following two criteria: (i) log2 fold-change ≥ 2 and (ii) false discovery rate (FDR) p-value correction of ≤ 0.05. Unsupervised hierarchical clustering was performed using the Expander 6.5.1^28^. The Pearson correlation was used as a distance measure for both row and column clustering and the clustering method was pairwise-average linkage. Corresponding *Arabidopsis thaliana* orthologues for all detected tobacco genes were determined by reciprocal best hits method.

The list of *Arabidopsis thaliana* orthologues was uploaded to ClueGO plug-in^29^ of Cytoscape^30^ for gene ontology analysis of topping and suckercide responsive genes. Enrichment analysis was based on a hypergeometric test. P-values were adjusted using Benjamini–Hochberg’s FDR; only FDR < 0.05 was considered as significant. For pathway analysis, a MapMan mapping file was specifically generated for the tobacco genes by the Mercator tool, which bins all genes according to hierarchical ontologies after searching a variety of databases and, finally, MapMan v.3.5.1 was used to visualize DEGs on different pathways^31^.

### Quantitative RT-PCR

Gene specific primers for the ten candidate genes were designed (Table S1) using Primer3 software^32^. RNA isolated from control, topped, FP- and OS-treated samples were reverse-transcribed using the Superscript III Reverse Transcriptase (Invitrogen, USA), following the manufacturer’s instructions. Quantitative PCR was performed as described by Pattanaik *et al.*^33^. All PCR reactions were performed in triplicate and repeated two times. The comparative cycle threshold (Ct) method (bulletin no. 2; Applied Biosystems, http://www.appliedbiosystems.com) was used to measure transcript levels. In addition to tobacco œ-tubulin (GenBank accession number AJ421411), tobacco elongation factor-1œ (GenBank accession number D63396) was also used as a reference gene.

## Results and Discussion

### DEGs in topped and suckercide-treated samples significantly overlap

Sequencing of RNA libraries from leaves of tobacco, that were un-topped, topped, or suckercide-treated post topping, generated a total of 120,598,443 reads (Fig. S1; Table S2). After removal of low quality reads, 99,930,676 high-quality reads were obtained. Each biological sample (un-topped, topped, or suckercide-treated post topping) was represented by an average of 23 million reads (Table S2). On an average more than 64% of the total reads from each condition were successfully mapped to the reference sequences (Table S2).

We identified 425 (350 upregulated and 75 downregulated), 384 (287 upregulated and 97 downregulated), and 274 (217 upregulated and 57 downregulated) DEGs in the leaves of topped, FP-, and OS-treated tobacco, respectively (Fig. S2; Tables S3–5). The numbers of upregulated genes were significantly higher in all three conditions compared to downregulated genes. Moreover, the number of DEGs was higher in FP-treated samples compared to those of OS-treated, indicating broader physiological and metabolic responses in FP-treated plants. Being a contact-localized-systemic suckercide, FP is absorbed when it comes in contact with plant tissues and is thus likely to affect more physiological and metabolic processes compared to OS, a contact suckercide.

**Figure 1:**
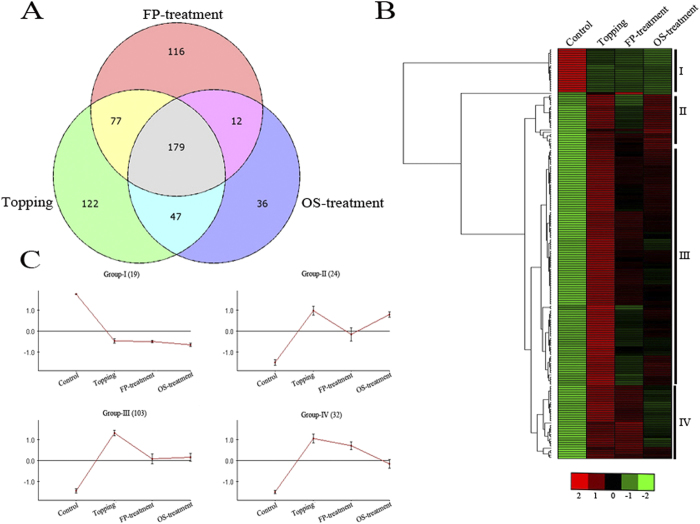
RNA-Seq analyses of differentially expressed genes (DEGs) in leaves of topped, FP- and OS-treated tobacco. (**A**) Venn diagram showing the overlap of DEGs between topped, FP-, and OS-treated samples. (**B**) Hierarchical cluster analysis and heatmap showing expression of common DEGs in topped, FP-, and OS-treated samples. (**C**) Expression profile of common DEGs associated with four different clusters.

We then compared the DEGs in topped and two suckercide-treated samples. The Venn diagrams showed that 179 DEGs were common to all three conditions (Fig. 1A). Although the number of DEGs varied among different samples, there was a high degree of overlap (42% for topping, 46% for FP-treatment, and 65% for OS-treatment) between all treatments. Only 29%, 30% and 13% of the total DEGs were unique to the topping, FP- and OS-treatment, respectively. Comparisons of the distributions of mean fold-change values for gene sets responsive to all treatments indicated that the magnitude of induction or repression of genes was higher in topping and lower in the suckercide treatments. Wilcoxon matched pairs test determined this difference was statistically significant (P < 0.0001).

Limited studies have been conducted on the influence of topping on gene expression patterns in tobacco. Previously, Qi *et al.* (2012) have used suppression subtractive hybridization to analyze topping responsive genes in tobacco roots. They have identified 129 DEGs which are largely related to secondary metabolism, defense response, and signal transduction^21^. We compared our datasets with the reported topping responsive DEGs. Both studies agree well on DEGs related to biosynthesis of phenylpropanoids and polyamines, as well as signal transduction.

### Topping and suckercide treatments differentially affect the expression of genes related to various metabolic and signaling pathways

To provide a more detailed overview of the expression patterns of genes responsive to topping and suckercide treatments, we performed hierarchical clustering analysis of the 179 DEGs common to all treatments. The analysis classified the DEGs into four groups with distinct expression profiles (Fig. 1B,C). DEGs in group-I (n = 19), which are mainly involved in photosynthetic processes, were repressed by both topping, and application of FP or OS (Fig. 1C). Expression of group II, III and IV genes, were significantly induced by topping, and repressed after suckercide treatments at different levels. Application of FP significantly repressed the expression of genes in group-II (n = 24), but the effect of OS was less pronounced. This group includes genes encoding lipase, kunitz family trypsin, and protease inhibitors, as well as those involved in the phenylpropanoid pathway (Fig. 1C). Group-III DEGs (n = 103), which are mainly related to wound response and biosynthesis of phenylpropanoids and their conjugates, constituted the largest group. FP and OS treatments downregulated the expression of wounding responsive genes, including those encoding defense-related proteins such as protease inhibitors, defensins, and lipid transferase, as well as regulatory proteins in the JA signaling pathway, such as jasmonate-ZIM-domain protein (JAZ) (Fig. 1C). DEGs in group IV (n = 32), while repressed by both FP and OS, were more strongly affected by OS than FP (Fig. 1C). Group-IV genes included those related to JA biosynthesis and wound signaling, such as *ALLENE OXIDE CYCLASE 3* (AOC3), *ALLENE OXIDE SYNTHASE* (AOS), and *PHOSPHOLIPASE D DELTA* (PLDDELTA). The roles of some of these genes in wounding response have been previously established. For example, wounding affects various aspects of lipid metabolism, including activation of phospholipase A and D, oxidation of fatty acids, and head-group acylation of monogalactosyldiacylglycerols^34^. Our data showed that the expression of *phospholipase D* was induced by topping, but repressed by the suckercides. Wounding-induced expression of *phospholipase D* has also been reported in castor^35^, cucumber^36^, and *Arabidopsis*^17^. Phospholipase D catalyzes the production of phosphatidic acid (PA), which acts as a precursor for JA synthesis^34^. In addition, PA is a signal mediator for wounding-induced activation of the mitogen-activated protein kinases (MAPK) pathway^18^. MAPKs are known to relay and amplify the signals through a reversible phosphorylation cascade in responses to phytohormones such as JA. Wound-inducible MAPK (WIPK) has been isolated and characterized for its role in JA signaling in tobacco^37^. Consistent with the previous report, we observed that the accumulation of WIPK transcripts was increased in response to topping; however, decreased following the application of suckercides (Fig. 1C).

### ClueGO analysis revealed significant enrichment of pathway genes involved in biosynthesis of JA, amino acids, and specialized metabolites

When using large sequencing data sets to assess the biological processes affected by topping and suckercide treatments, it is necessary to connect the DEGs with gene ontology (GO) and biological pathways. We used ClueGO, a Cytoscape plug-in, to generate a GO/pathway term network. ClueGO integrates GO terms with Kyoto Encyclopedia of Genes and Genomes (KEGG) pathways, to create functionally organized GO category networks^29^. ClueGO analysis of 425, 384 and 274 DEGs in topped, FP- and OS-treated samples, respectively, showed that the downregulated genes in topping and suckercide treatments were largely associated with proteins targeted to chloroplasts (Fig. S3–5). Pathogen or herbivore attack significantly affects the expression of genes related to the photosynthetic machinery of plants^38^. Bilgin *et al.* (2010) compared the transcriptome data from 22 different biotic damages on eight plant species and reported that transcript levels of 84% of the genes with chloroplast targeting peptide sequences, were decreased^39^. In tomato, expression of 10–12% of the genes related to photosynthesis were downregulated within 1 to 8 h after wounding^40^. Our results are consistent with previous findings and support the notion that reduction of photosynthetic proteins allows plants to invest more resources into immediate defense responses after wounding or topping in order to survive in adverse conditions. As expected, GO terms related to phenylpropanoid biosynthetic process, wound response, and JA biosynthesis were enriched in upregulated genes (Fig. S3–5). Phenylpropanoids are natural compounds derived from the amino acid phenylalanine, and their role in plant defense is well documented. They act as chemical barriers against biotic and abiotic stress, including wounding, and also function as signal molecules involved in local and systemic signaling^41^. JA is an important mediator of wound signaling. *Arabidopsis* mutants, insensitive to JA, showed reduced wound response^42^. Interestingly, the GO term ‘aspartate family amino acid metabolic process’ was also enriched in all conditions. This GO term includes the metabolism of amino acids of the aspartate family, including aspartate, asparagine, lysine, threonine and methionine. These amino acids provide a substantial carbon pool for stress-related catabolism to feed the tricarboxylic acid cycle in various organisms. Furthermore, the catabolism of these amino acids has been reported to be stress inducible^43^. These observations suggest that as the photosynthetic activity is reduced after topping, plants utilize the stored carbon source to meet the requirement of energy-intensive production of secondary metabolites. The enrichment of ‘tryptophan catabolic process’ in FP-treatment was another interesting finding. Tryptophan acts as a precursor of auxin biosynthesis in plants^44^. Induction of tryptophan catabolic genes probably results in increasing accumulation of auxin in response to FP-treatment, leading to inhibition of sucker formation.

**Figure 2:**
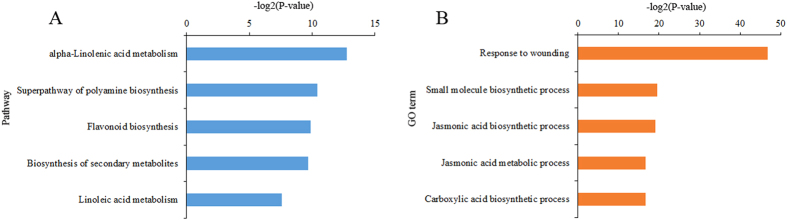
Pathway and Gene ontology (GO) analysis of DEGs common in topped, FP- and OS-treated samples. (**A**) Five significant pathways associated with DEGs common in topped, FP-, and OS-treated samples. The vertical axis represents the pathway category, and the horizontal axis represents the -log2 (P-value) of the pathways. Greater -log2 (P-value) scores correlated with increased statistical significance. (**B**) Five significant GO terms (biological processes) associated with the DEGs common in topped, FP-, and OS-treated samples. The vertical axis represents the GO category, and the horizontal axis represents the -log2 (P-value) of the significant GO terms.

Biological functions and pathways associated with the 179 DEGs common to topping and suckercide treatments were also investigated (Fig. 2A,B). Pathway analyses of the common DEGs revealed that biosynthetic pathways of linolenic acid and linoleic acid are among the most affected (Fig. 2A). Lipid-based signaling, via the octadecanoid pathway, leads to the production of the key signal molecule, JA, in plant wounding response. Linolenic acid and linoleic acid are present in low amounts in healthy and intact plant cells, but their levels increase in both cultured cells as well as plants subjected to wounding or elicitors^45^,^46^. Linolenic acid is also a precursor for JA biosynthesis. Biosynthesis of specialized metabolites, such as polyamines and flavonoids, are also affected by all treatments (Fig. 2A). Wounding response, small molecule biosynthesis, and JA metabolism, were the most affected biological processes, in response to topping, FP- and OS-treatments (Fig. 2B). Our observations are consistent with previous findings and emphasize the importance of these biological pathways/processes in topping/wounding response.

### Topping and FP treatment affect hormone and wound signaling, as well as DNA repair and replication

**Figure 3:**
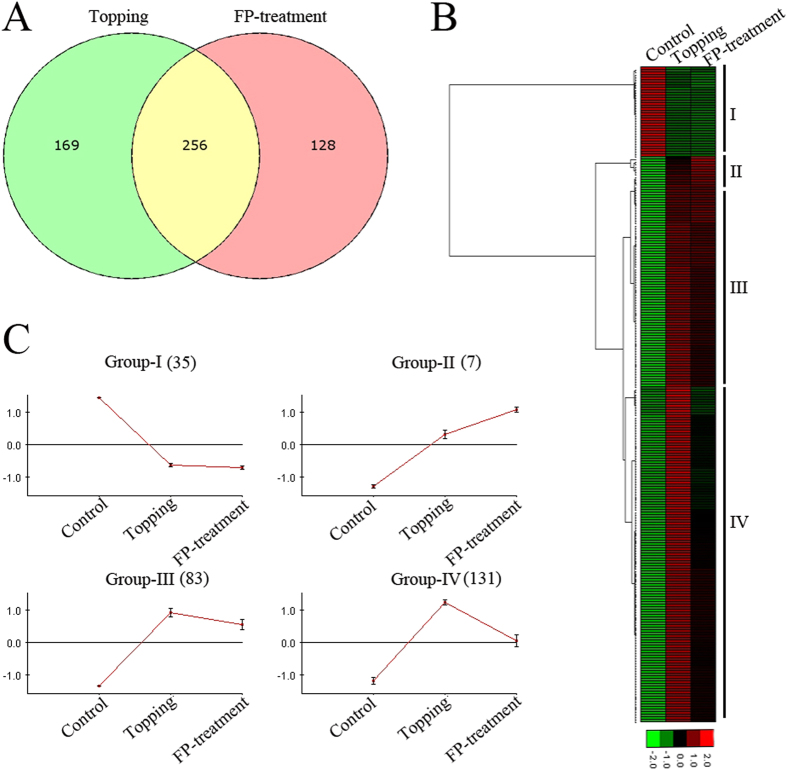
RNA-Seq analyses of DEGs in leaves of topped and FP-treated tobacco. (**A**) Venn diagram showing the overlap of DEGs between topping and FP-treatment. (**B**) Hierarchical cluster analysis and heatmap showing expression of common DEGs in topped and FP-treated leaves. (**C**) Expression profile of common DEGs associated with four different clusters.

We next compared the DEGs between topping and FP-treatment. The Venn diagram analysis (Fig. 3A) showed that approximately 60% of the genes differentially expressed in response to topping were common to those affected by FP-treatment. We performed hierarchical clustering analysis to generate an overview of the expression patterns of the 256 DEGs common to topping and FP treatment (Fig. 3B). This analysis classified the 256 DEGs into four groups (Fig. 3B,C) based on their expression profiles. DEGs in Group-I (n = 35), which are mostly related to photosynthetic processes, were repressed in response to both topping and FP-treatment (Fig. 3C; Table S6A). DEGs in group-II (n = 7), which are largely related to DNA replication and repair, showed increased expression in both conditions (Fig. 3C; Table S6A). Wounding of plants trigger the activation of genes related to defense as well as the reconstruction of damaged tissues. Upregulation of DNA replication and repair genes suggests a possible role in cell differentiation and healing processes in plants. Group-III (n = 83) and Group-IV (n = 131) together constitute about 83% of the DEGs that were induced in response to topping, and repressed following FP-treatment; however, the magnitude of induction or repression differed in the two groups (Fig. 3C). DEGs in group-III and group-IV are largely related to JA biosynthesis (*e.g.* AOC3, AOS) and signaling (*e.g.* MPK3, JAZ3), fatty acid metabolism, and aromatic compound metabolism (*e.g.* MYB15 and NtMYBJS1). JAZ proteins are the key components in the JA signaling pathway. They interact with JA-responsive regulatory proteins to form a repressor complex that diminishes the expression of downstream target genes. Upon perception of the JA signal, JAZ proteins are degraded by the 26 S/ubiquitin proteasomal system^47^. The regulators are then released to activate the transcription of downstream genes. MYB15 is a R2R3-MYB transcription factor (TF) which has been functionally characterized in *Arabidopsis*^48^ and grape^49^. Overexpression of *MYB15* confers drought and salt tolerance to *Arabidopsis*, whereas in grapes it induces the biosynthesis of a small group of phenylpropanoids, stilbenes, known to play a role in plant defense. NtMYBJS1 is a JA-inducible R2R3-MYB that regulates the expression of several phenylpropanoid pathway genes, including *phenylalanine ammonia-lyase* (PAL) and *4-coumarate:CoA ligase* (4CL) and induced the accumulation of phenylpropanoid-polyamine conjugates in tobacco^50^. In our data set, topping induced, but FP application, repressed, the expression of both MYB15 and NtMYBJS1. TFs often regulate an entire pathway in response to biotic or abiotic cues. The counter effects of topping and FP treatment on TFs, such as MYB15 and NtMYBJS1, likely represent a general mechanism for metabolic reprogramming in decapitation and suckercide treatments.

In addition, topping and FP-treatment differentially regulated the expression of a homolog of *Arabidopsis* MYB13, a TF that is possibly involved in shoot morphogenesis in plants. In *Arabidopsis*, MYB13 transcripts accumulate in response to wounding, exogenous ABA treatment, and dehydration. Ectopic expression of MYB13 alters branching pattern of inflorescence in *Arabidopsis*^51^. Expression of the tobacco *MYB13* was induced in response to topping; however it declined significantly after application of FP.

Furthermore, topping and FP application altered the expression of genes encoding amidohydrolase, which are homologous to *Arabidopsis IAA-ALANINE RESISTANT 3* (*IAR3*), and *IAA-LEUCINE RESISTANT-LIKE GENE 2* (*ILL2*) and *ILL6*. Expression of *ILL6* was increased further after FP application. Recently, a homologue of *IAR3*, *JASMONOYL-L-ISOLEUCINE HYDROLASE* 1 (*JIH1*), has been isolated and characterized for its role in regulation of JA-Isoleucine (JA-Ile) levels in *Nicotiana attenuata*^52^. Phytohormones can be accumulated in free forms, or conjugated to amino acids or sugars. The conjugation and de-conjugation are dynamic processes that modulate the levels of active phytohormones in plants. IAA becomes biologically inactive after conjugation to sugars or amino acids. In contrast, JA-amino acid conjugate (e.g. JA-Ile) is the bioactive form that binds to the JA co-receptor CORONATINE INSENSITIVE1 (COI1), and promotes JA signaling. The amidohydrolases, IAR3,ILL2 and ILL6 have been shown to cleave the auxin- and JA-amino acid conjugates and regulate their turn over in plants^53^,^54^ Topping-induced expression of genes encoding three amidohydrolases suggests that they are probably involved in the turnover of JA and IAA in response to topping and suckercide treatments. It is possible that the hydrolysis rates of JA- and IAA conjugates are different in topped or FP-treated plants. Upregulation of *ILL6* in FP treated plants probably results in the accumulation of higher levels of biologically active IAA that possibly influences cytokinin level and inhibits sucker development.

To gain insights into FP specific responses, we divided the 128 genes unique to FP-treatment, based on up- or down-regulation (Table S6B). GO term enrichment analysis revealed that the majority of downregulated genes were related to plastid organization and nitrogen compound metabolism, while upregulated genes were associated with wound and defense responses. Upregulated genes include *PHYTOSULFOKINE 3 PRECURSOR* (PSK3), *JASMONATE-REGULATED GENE 21* (JRG21), and *RESPIRATORY BURST OXIDASE HOMOLOGUE D* (RBOHD). Phytosulfokines (PSKs) are sulfated pentapeptides encoded by small gene families, and present both in monocots and dicots. PSKs have recently been implicated in wound response in *Arabidopsis*^55^. Plant respiratory burst oxidases (RBOHs) are homologous to human neutrophil pathogen-related gp91^phox^, and have been cloned from a number of plant species, including tobacco, *Arabidopsis* and tomato. They are required for reactive oxygen species (ROS) accumulation in plant defense responses^56^,^57^,^58^. Our results suggest that FP attenuates the effect of topping by repressing the expression of some wounding inducible genes while inducing a new set of wounding and defense related genes.

In addition, FP-treatment also affected the expression of genes shown to be involved in cell dedifferentiation in other plants. WOUND INDUCED DEDIFFERENTIATION 1 (WIND1), an AP2/ERF TF, is rapidly induced at the wounding site, and controls cell dedifferentiation in *Arabidopsis*. Wounding also induces the B-type ARABIDOPSIS RESPONSE REGULATOR (ARR)-mediated cytokinin response, and WIND1 acts via ARR-dependent signaling pathway, to promote cell dedifferentiation in *Arabidopsis*^59^. The expression of a tobacco homolog of *AtWIND1*, did not change significantly in response to topping; however, it was repressed after FP-treatment, suggesting that FP probably represses cytokinin signaling and inhibits cell differentiation in plants.

### OS-treatment affects the expression of genes related to specialized metabolism, JA signaling, and water stress

**Figure 4:**
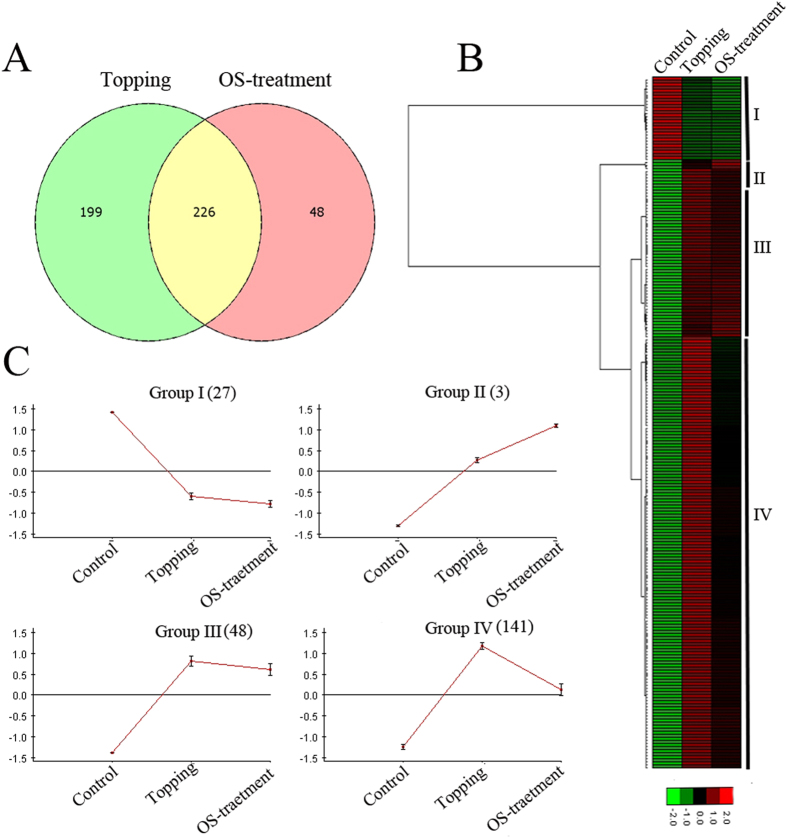
RNA-Seq analyses of DEGs in leaves of topped and OS-treated tobacco. (**A**) Venn diagram showing the overlap of DEGs between topping and OS-treatment. (**B**) Hierarchical cluster analysis and heatmap showing expression of common DEGs in topped and OS-treated leaves. (**C**) Expression profile of common DEGs associated with four different clusters.

We also compared DEGs common to topping and OS-treatment. The Venn diagram (Fig. 4A) showed that 82% of the genes, differentially expressed in OS-treated samples, were common in topping. Hierarchical clustering analysis classified the 226 DEGs common to topping and OS-treatment into four distinct expression profiles (Fig. 4B,C). DEGs in Group-I (n = 27) were repressed in response to both topping and OS-treatment. Similar to FP-treated samples, Group-I was enriched in genes related to photosynthesis and light responses (Table S7A). Expression of genes in group-II (n = 3) was induced by both conditions. Groups-III (n = 48) and IV (n = 141) were induced by topping, but repressed by OS-treatment; however, the degrees of induction or repression were different in the two groups (Fig. 4C). Group III and IV genes affected by OS-treatment include *PHENYLALANINE AMMONIA-LYASE 2* (*PAL2*), and *GERANYLGERANYL REDUCTASE* (*GGR*), *LOX6*, *PLDDELTA*, *AOC3*, *JAZ3*, *JAZ2*, and several transporters, such as *OLIGOPEPTIDE TRANSPORTER 7* (*OPT7*) and *CHLOROQUINE-RESISTANCE TRANSPORTER-LIKE TRANSPORTER 2* (*CLT2*). These genes are largely related to phenylpropanoid and small molecule biosynthetic processes, JA signaling, cellular amino acid metabolism, and coumarin metabolism (Table S7A). Coumarins, lactones of phenylpropanoids, are active allelochemicals widely distributed in the plant kingdom, and are important for plant–plant interaction and communication^36^.

To investigate the OS specific responses further, we divided the 48 genes unique to OS-treatment, based on up- or down-regulation (Table S7B). GO term enrichment analysis revealed that most of the downregulated genes were related to plastid organization and photosynthesis, while most of the upregulated genes were related to water deficit responses. Several genes related to water stress, including *DEHYDRIN XERO 1*, *GLUTATHIONE S-TRANSFERASE TAU 19* (*GSTU19*) and *HOMEOBOX 7* (*HB7*), were highly upregulated in response to OS-treatment. In *Arabidopsis*, the expression of *HB7*, which encodes a homeodomain leucine zipper TF, was reported to be induced by water deficit, osmotic stress, and exogenous treatment with abscisic acid (ABA)^60^. In our data set, *HB7* was upregulated after OS application. Induction of water deficit related genes supports the assumption that OS application results in plasmolysis and water loss, leading to the death of young axillary shoots^11^.

### MapMan visualization highlights the influence of topping and suckercide treatments on different plant metabolic pathways

**Figure 5:**
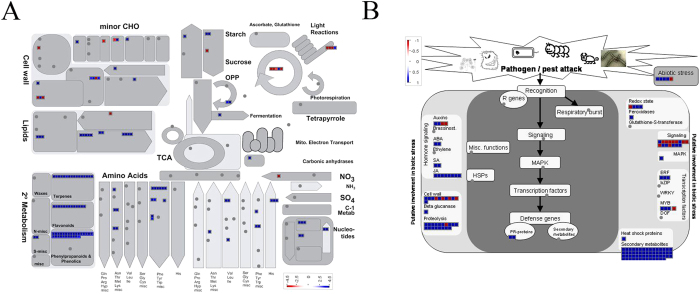
MapMan visualization of differential gene expression in topped leaves compared with control. Blue denotes up-regulation and red down-regulation. The log2 fold changes of significantly DEGs were imported and visualized in MapMan for the topped tobacco leaves sample with regard to a metabolism overview (**A**) and pathogen/pest attack (**B**).

**Figure 6:**
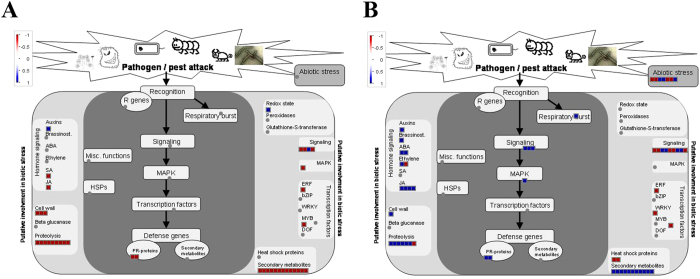
MapMan visualization of differential gene expression in FP-treated leaves compared with topped leaves. Blue denotes up-regulation and red down-regulation. The log2 fold changes of significantly DEGs, common in topped and FP-treated tobacco leaves (**A**) and unique to FP-treated leaves (**B**), were imported and visualized in MapMan with regard to pathogen/pest attack.

Pathway-based analysis intends to associate biological functions with genes differentially expressed in response to various biotic and abiotic factors. We used a comprehensive tool, the MapMan, to visualize the pathways affected by topping and suckercide treatments in tobacco. We overlaid the log2 fold change of DEGs to identify affected pathways. Consistent with the GO analysis, DEGs related to secondary metabolite biosynthesis were upregulated in response to topping (Fig. 5), but repressed by the application of FP or OS (Fig. 6A; Fig. S6A). Also in agreement with the GO analysis, the JA pathway was highly activated by topping, but significantly repressed by FP treatment (Figs 5B and 6A, Fig. S6A). Other phytohormones, including auxin, abscisic acid (ABA), salicylic acid (SA) and ethylene, did not exhibit such opposite responses. Genes related to proteolysis and signaling, were also affected by topping and suckercide treatment, to varying degrees (Figs 5B and 6; Fig. S6). Proteolysis is a crucial metabolic process essential for protein processing and turnover. The plant genome harbors large proteolytic machinery that involves irreversible degradation of proteins. At least 826 protease genes have been identified in *Arabidopsis*. This machinery acts as a housekeeper by removing non-functional proteins and recycling amino acids, and also as a protector by regulating biological processes, such as the recognition of pathogens and the induction of defense responses^61^. However, proteases can potentially be harmful to plants, if present in higher levels. The activities of proteases are strictly regulated by a group of proteins, proteinase inhibitors (PINs). PINs are also known to act on proteolytic enzymes secreted by herbivorous insects and microorganisms, and play a role in plant defense. Topping induced the expression of several classes of PINs, including PIN II and cysteine PIN I, which are involved in plant defense^17^,^62^. However, application of FP reduced the expression of topping inducible protease genes (Figs 5B and 6A). In addition, topping induced the expression of genes encoding a number of TF protein families, including R2R3MYBs, which are known to be involved in secondary metabolite biosynthesis, as well as plant growth and development. Application of FP, on the other hand, repressed the expression of these regulatory genes.

Next we chose the 128 unique DEGs of FP-treatment and mapped the log2 fold change on the different pathways. As shown by GO analysis (Fig. S4), FP-treatment affected the expression of a unique set of genes related to wounding and metabolism of phytohormones, including JA and IAA (Fig. 6B). Moreover, expression of several classes of TF genes, including ERFs and WRKYs, were repressed specifically in response to FP-treatment. WRKY TFs are defined by the highly conserved amino acid sequence, WRKYGQK, and involved in the regulation of numerous biological processes in plants. Several members of this family, including WRKY11, WRKY17 and WRKY70, play critical roles in the antagonistic interaction between SA and JA^63^,^64^. FP-treatments resulted in significant downregulation of *WRKY70*, compared to the control. FP-treatment also repressed the expression of *ERF* known to be involved in the regulation of cell dedifferentiation in plants^59^. The log2 fold changes of DEGs unique to OS treatment were also mapped on the different pathways. Genes related to abiotic stress and proteolysis were downregulated whereas some genes related to secondary metabolism, cell wall and JA were upregulated (Fig. S6B).

### Quantitative RT-PCR analysis of selected DEGs validates the RNA-seq data

**Figure 7:**
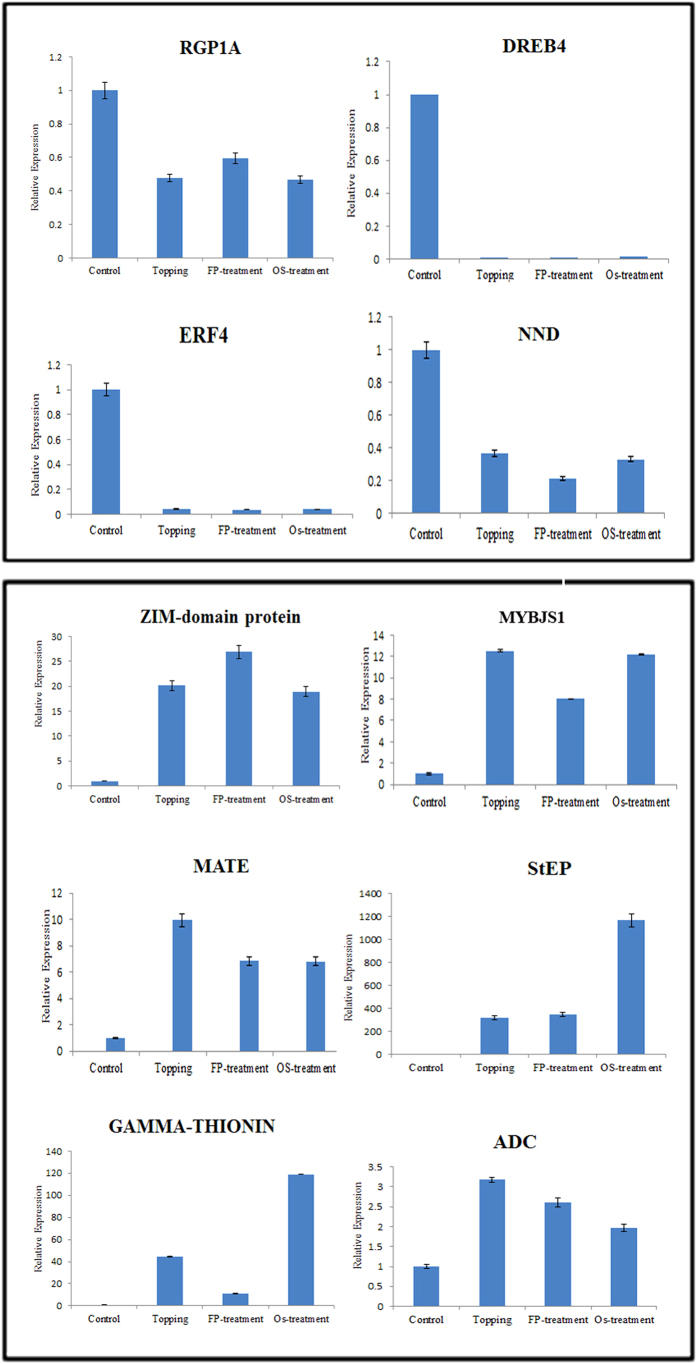
Validation of deep sequencing results using quantitative real-time polymerase chain reaction (qRT-PCR) analysis. The tobacco œ-tubulin was used as an internal control for normalization. Data represent mean ± SD of three independent replicates.

To validate the RNA-seq results, expression of a selected set of six upregulated and four downregulated genes were analyzed by qRT-PCR (Fig. 7). These genes encode the MATE efflux family protein (MATE), Stigma Expressed Protein (StEP), JAZ factor, Gamma Thionin, Dehydration-Responsive Element-Binding protein 4 (DREB4), Ethylene-Responsive Factors (ERFs), JA-induced MYB ( MYBJS1), nicotine N-demethylase (NND), arginine decarboxylase (ADC), and RNA-binding Glycine-rich Protein-1a (RGP1a). The qRT-PCR results complemented the RNA-seq data, confirming the reliability and accuracy of our RNA-seq in this study.

## Conclusion

Our findings provide insights into global changes in gene expression pattern in biological pathways responsive to topping and suckercide treatments in tobacco plants. The identification and functional annotation of DEGs revealed that topping and suckercide treatment had significant effect on primary as well as secondary metabolic pathways in plants.

**Figure 8:**
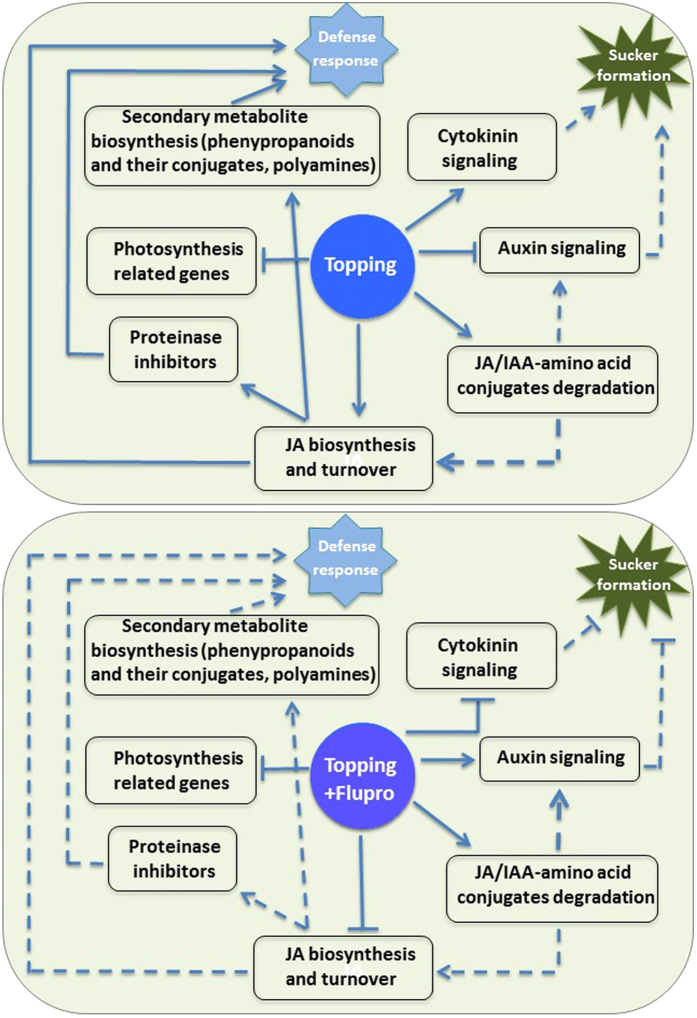
A hypothetical depiction of plant responses to topping and FP-treatment based on the differential gene expression patterns. The upper panel shows the major effects of topping on key biological processes, and the lower panel shows the effects of FP-treatment. Arrows represent positive regulation; T-bars indicate negative regulation. Dashed lines represent possible regulation through in-direct or combined effects of up- or down-regulated genes.

Based on our findings and data available in the literature, we have developed a hypothetical model that depicts the biological processes affected after topping and the application of suckercide (FP) post topping (Fig. 8). JA is known as the major regulator of wound signaling in plants. Topping induces JA biosynthesis, leading to the upregulation of genes related to proteinase inhibitors and secondary metabolite biosynthesis. Higher expression of aminohydrolase genes, in response to topping, probably helps maintain JA homoeostasis in plants. In addition, topping positively influences cytokinin signaling, and promotes axillary shoot proliferation. Suckercide, *e.g.* FP, application possibly attenuates the effects of topping, which in turn, affects JA and secondary metabolite biosynthesis. Suckercides also repress the expression of genes potentially involved in cytokinin signaling. Moreover, higher expression of the aminohydrolase genes probably results in increased hydrolysis of auxin-amino acid conjugates, leading to the accumulation of more biologically active auxin, which inhibits sucker formation.

Application of suckercides leaves undesirable chemical residues on plants and results in pollution of soil and water. In addition, application of suckercides is expensive and requires specific machinery. Furthermore, systemic suckercides, such as MH, are potentially carcinogenic^65^. We have identified several topping-inducible genes that are highly upregulated in response to topping. Regulatory genes controlling axillary shoot development have been identified and characterized in a number of plant species including *Arabidopsis*, tomato and capsicum^66^,^67^,^68^. Up or down regulation of these genes by biotechnological means in a topping-inducible manner may prevent sucker formation without the need for suckercide application. Promoter elements of topping-inducible genes can potentially be used to develop a chemical-free sucker control system by altering the expression of genes related to axillary shoot formation only after topping, thus circumventing negative effects on plant development.

## Additional Information

**How to cite this article**: Singh, S. K. *et al.* RNA-sequencing Reveals Global Transcriptomic Changes in *Nicotiana tabacum* Responding to Topping and Treatment of Axillary-shoot Control Chemicals. *Sci. Rep.* **5**, 18148; doi: 10.1038/srep18148 (2015).

## Supplementary Material

Supplementary Information

Supplementary Table S3

Supplementary Table S4

Supplementary Table S5
