# Supplementary material for: RNA-sequencing Reveals Global Transcriptomic Changes in Nicotiana tabacum Responding to Topping and Treatment of Axillary-shoot Control Chemicals
Source: Sci Rep. 2015 Dec 16;5:18148. doi: 10.1038/srep18148 (PMC4680964; doi:10.1038/srep18148)
Supplement: Supplementary Information [file srep18148-s1.pdf]

Supplementary Information

RNA-sequencing Reveals Global Transcriptomic Changes in *Nicotiana tabacum* Responding to Topping and Treatment of Axillary-shoot Control Chemicals

Sanjay K. Singh, Yongmei Wu, Jayadri S. Ghosh, Sitakanta Pattanaik, Colin Fisher, Ying Wang, Darlene Lawson, and Ling Yuan

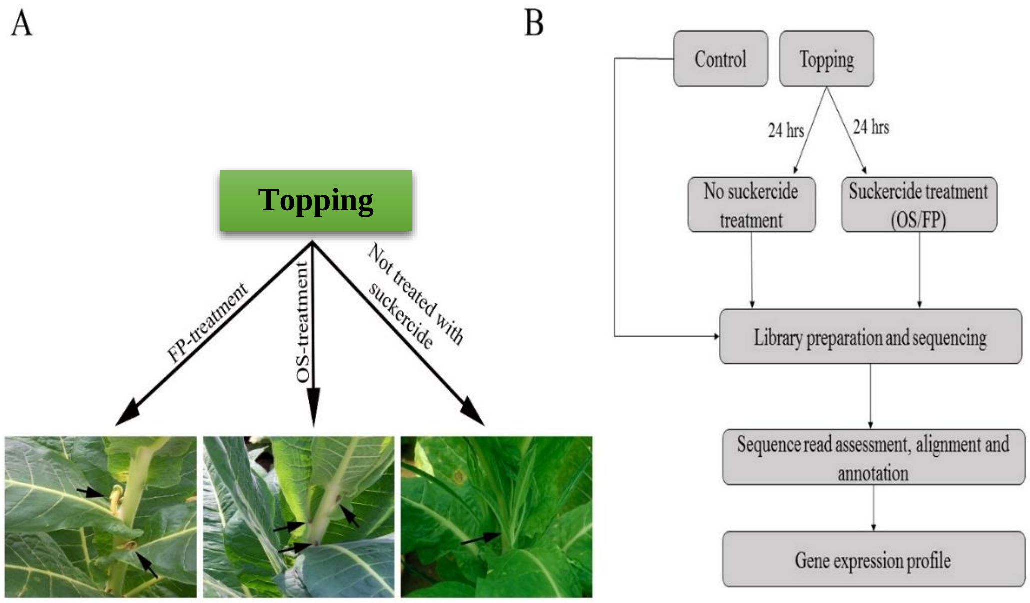

Figure S1. Schematic diagram of the experimental procedure. (A) Effect of topping and suckercide (OS and FP) treatment. (B) Experimental design for topping and suckercide treatment and gene expression analysis.

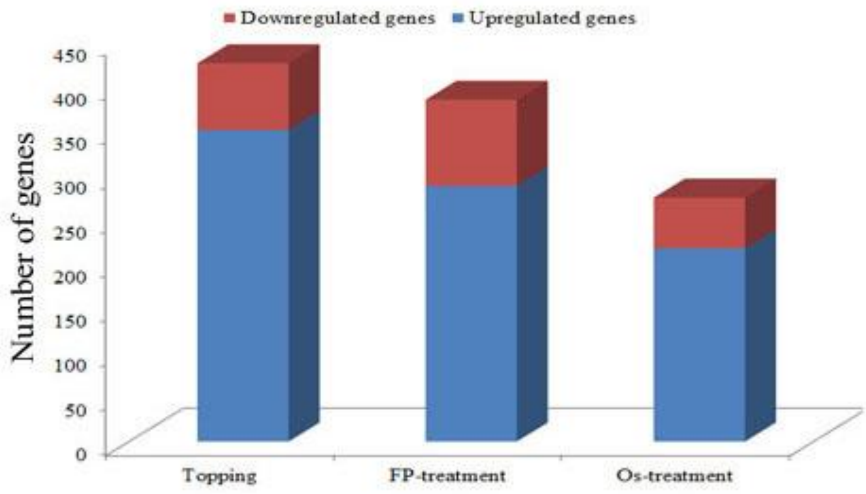

Figure S2: Numbers of upregulated and downregulated differentially expressed genes (DEGs) in topped, FP- and OS-treated samples.

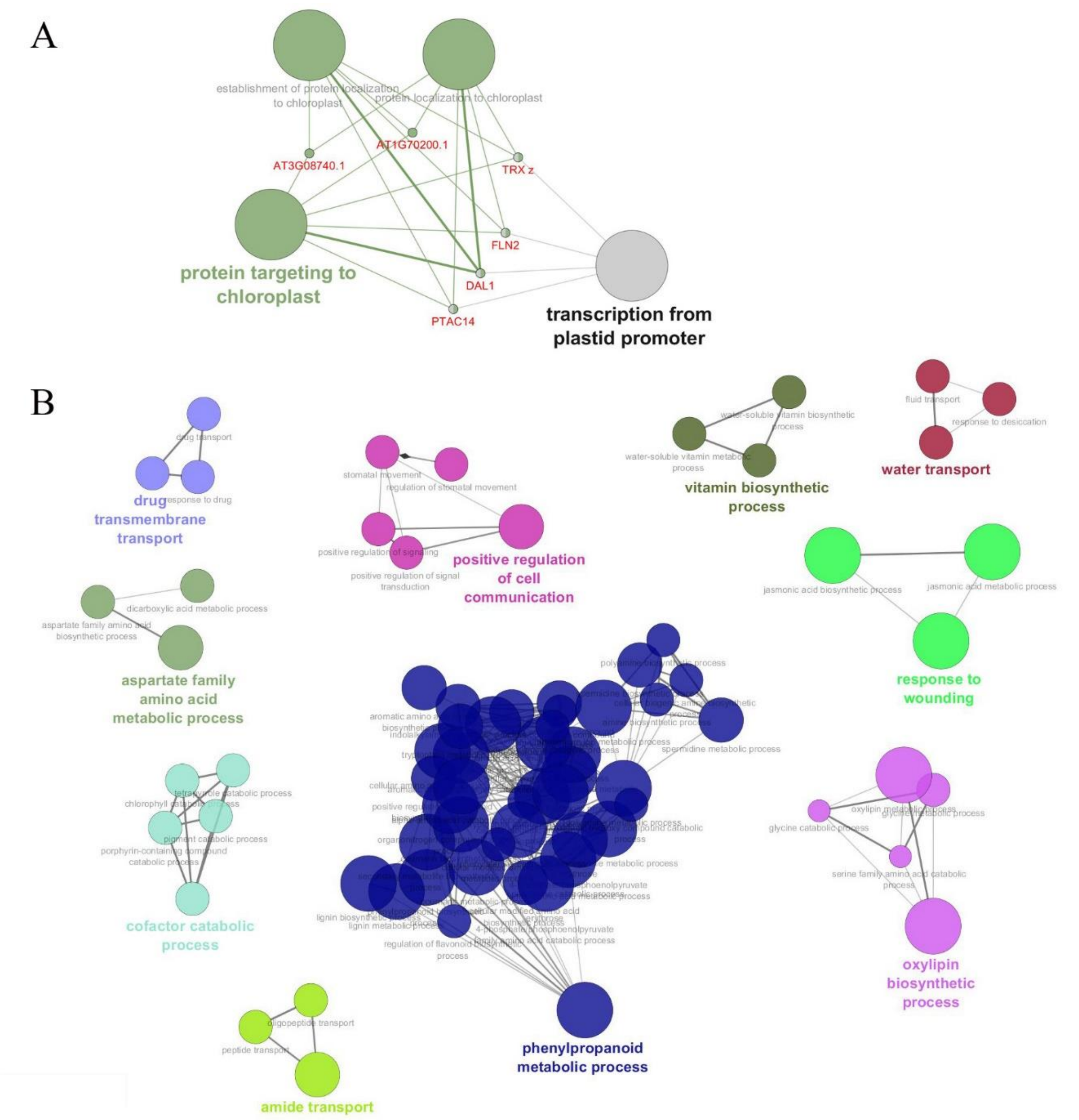

Figure S3. ClueGO network analysis. Analysis shows significant regulation of several GO categories involved in topping response in tobacco leaves. The nodes represent significantly changed GO categories. Lines represent the overlap between different categories. All nodes with a large overlap have a similar color. (A) Downregulated genes (B) Upregulated genes

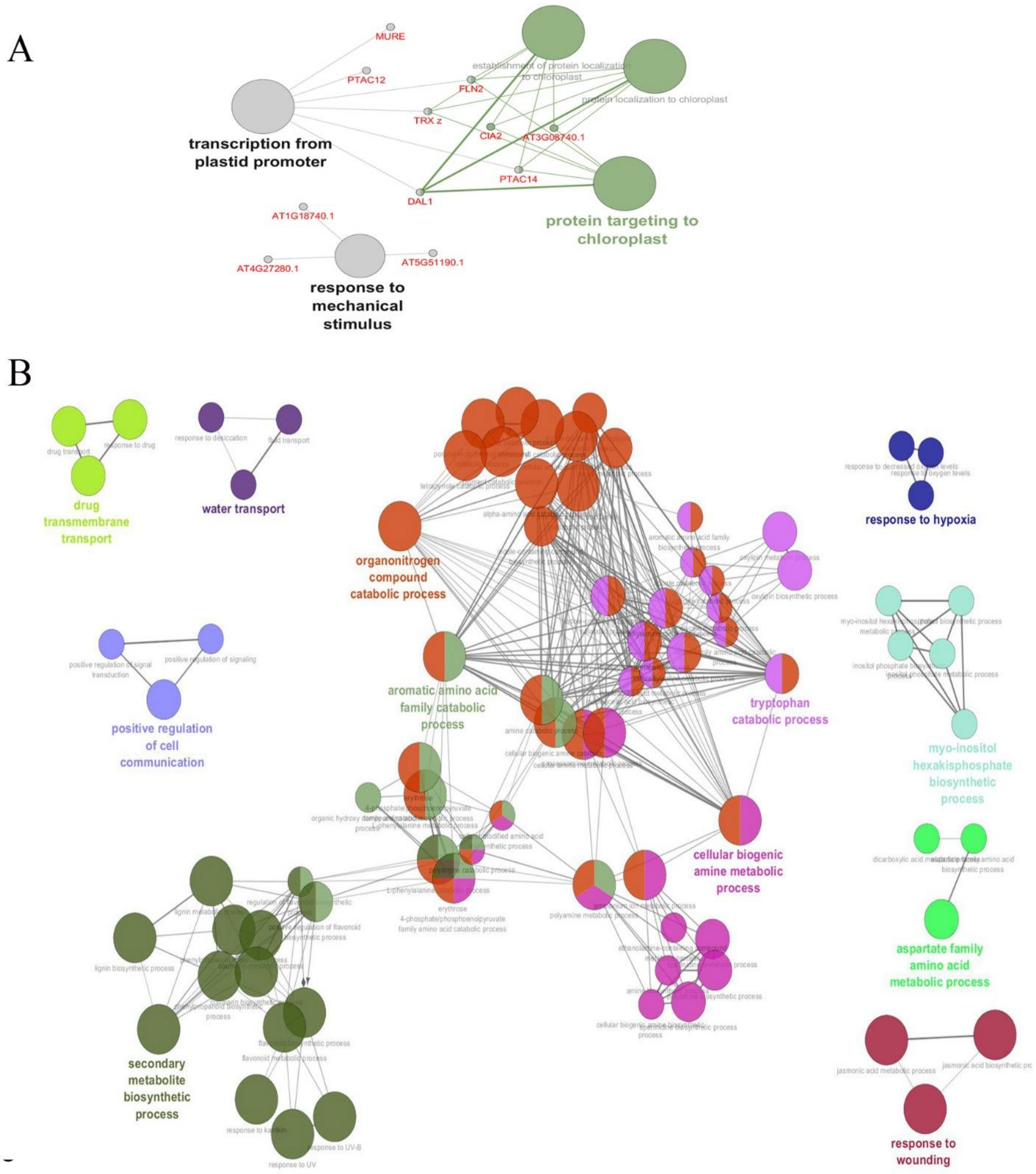

Figure S4. ClueGO network analysis. Analysis shows significant regulation of several GO categories involved in FP response in tobacco leaves. The nodes represent significantly changed GO categories. Lines represent the overlap between different categories. All nodes with a large overlap have a similar color. (A) Downregulated genes (B) Upregulated genes

A

B

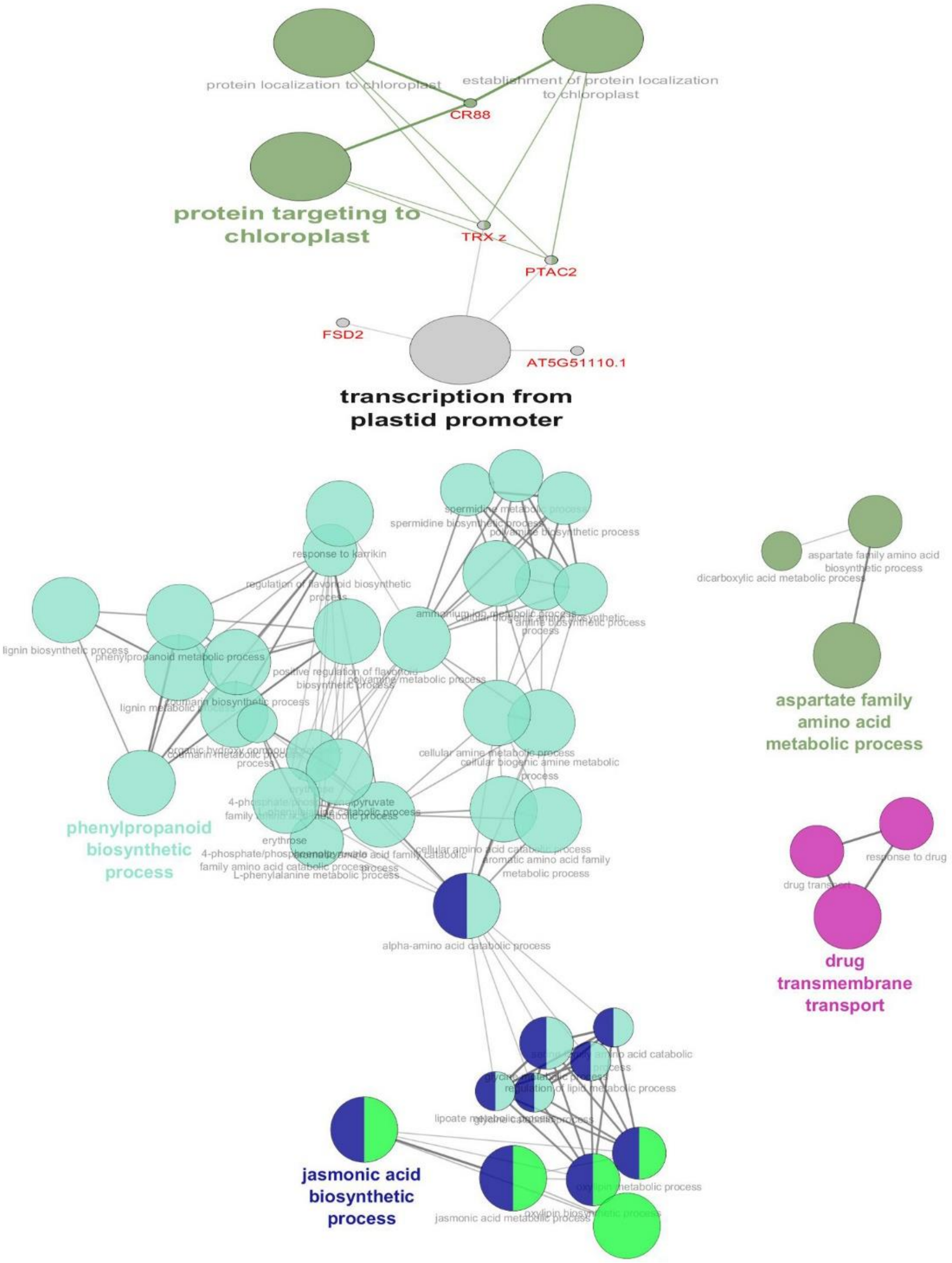

Figure S5. ClueGO network analysis. Analysis shows significant regulation of several GO categories involved in OS response in tobacco leaves. The nodes represent significantly changed GO categories. Lines represent the overlap between different categories. All nodes with a large overlap have a similar color. (A) Downregulated genes (B) Upregulated genes

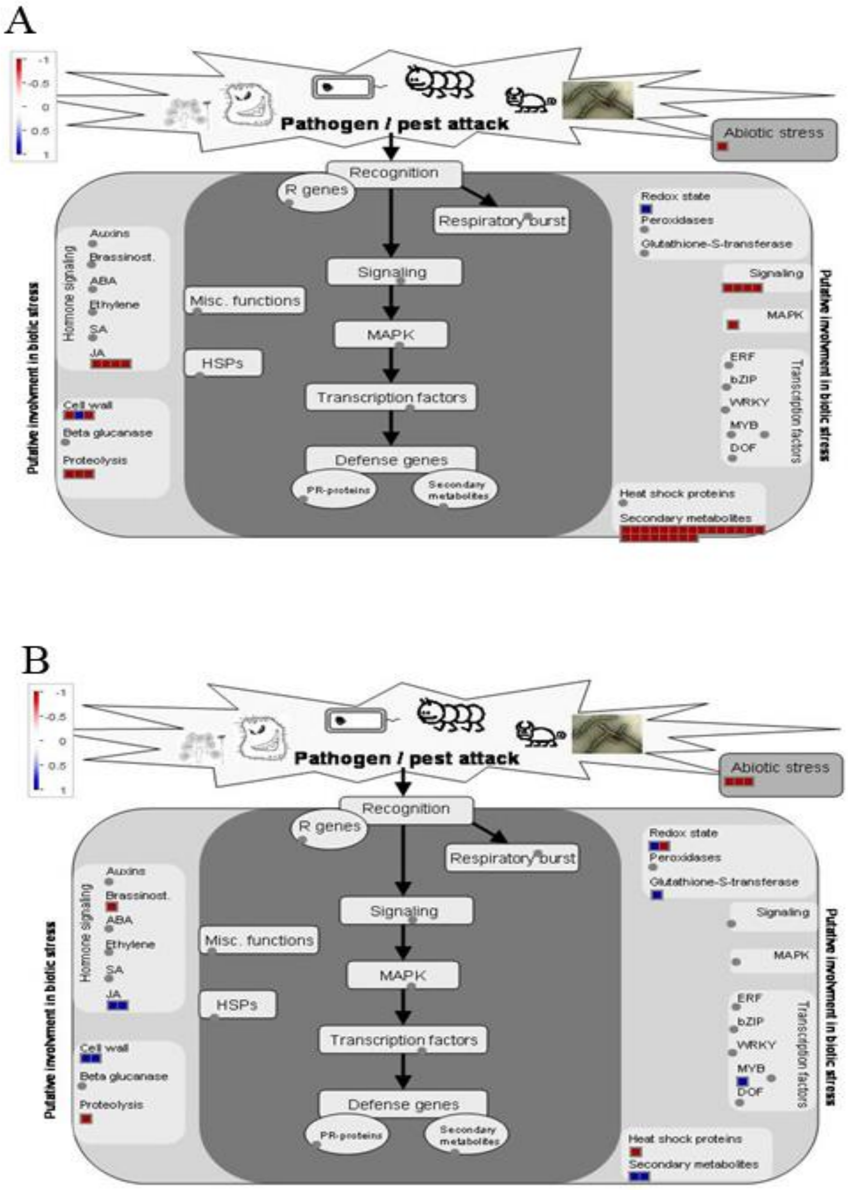

Figure S6. MapMan visualization of differential gene expression in OS-treated tobacco leaves compared with topped tobacco leaves. Blue denotes up-regulation and red down-regulation. The log2 fold changes of significantly DEGs common in both topped and OS-treated tobacco leaves (A) and unique to OS-treated tobacco leaves (B) were imported and visualized in MapMan with regard to pathogen/pest attack.

Table S1: The list of the primers used to conduct RT-qPCR analysis

Gene name	Forward primer	Reverse primer
**RGP1A**	5′-ACACTTGGGGAGGCTTTTTC-3′	5′-ATAGCGTCCCTCATGGCTTTC-3′
**DREB**	ACTTTGCTGATTCGGCTTGG	AATGTTTCGGCCGCTTCAAC
**ERE4**	5′-AGCTTCAAAGAACGCAAGCC-3′	5′-ATTTCTGCTGCGAACTTGCC-3′
**NND**	5′-TTTACGGCGAATACCTTGGC-3′	5′-TGGCGAATCTCACGTGTTTG-3′
**ZIM-domain protein**	5′-TGCGACAATGAAGCAGCAAC-3′	5′-TTCAGTTATCCCAGCCACAGAG-3′
**MYBJS1**	5′-AGGTGCGGAAAAAGTTGTCG-3′	5′-TTGCTGCAATTCCAGACCAC-3′
**MATE**	5′-TGGCCTGGATTGGTTTTGTG-3′	5′-AGCTATTGCATTGGCCCAAC-3′
**StEP**	5′-TCCACCAATCCCATTGTCCTTC-3′	5′-GCGGTTTGTCGTTTATGTCTAGC-3′
**Thio**	5′-AGGTGCATTTGCTACAAGCC-3′	5′-AGCTGCAGCCAAAGTTTCTG-3′
**ADC**	5’-ATTTGGGCTCACGACAACAC-3’	5’-AACGCCATCAGCAAGCAAAG-3’
**TUB**	5’-GGTATTCAGGTCGGAAATGC-3’	5’-ATCTGGCCATCAGGCTGAAT-3’
**EF**	5’- ACCACTGGTGGTTTTGAAGC-3’	5’- ACACCAAGGGTGAAAGCAAG-3′

Sample name*	Raw reads	Clean reads	GC content (%)	Mapped reads (%)
Control_1	16,653,396	14,007,366	49	71.40
Control_2	12,755,566	10,732,758	46	73.20
Topping_1	17,939,985	14,503,970	47	67.01
Topping_2	15,333,062	13,614,869	50	61.75
FP-treatment_1	13,894,395	12,084,450	49	86.06
FP-treatment_2	13,322,332	11,663,364	47	79.47
Os-treatment_1	13,126,659	11,433,606	50	82.53
Os-treatment_2	17,573,048	11,890,293	47	83.44

Table S2: Summary of RNA-seq and mapping results.

*The numbers 1 and 2 at the end of the sample name represent replicate number of the same library

Table S6. Summary of the GO term enrichment analysis of (**A**) common DEGs in Topping and FP-treatment (**B**) FP-treatment specific DEGs

A

GO-ID	p-value	Description
**Group-I (35)**		
42793	4.84E-07	transcription from plastid promoter
45036	4.84E-07	protein targeting to chloroplast
9058	1.35E-05	biosynthetic process
6399	1.60E-05	tRNA metabolic process
6364	1.86E-05	rRNA processing
**Group-II (7)**		
731	1.78E-04	DNA synthesis involved in DNA repair
76	5.35E-04	DNA replication checkpoint
30174	5.35E-04	regulation of DNA-dependent DNA replication initiation
32297	5.35E-04	negative regulation of DNA-dependent DNA replication initiation
6269	7.13E-04	DNA replication, synthesis of RNA primer
**Group-III (83)**		
9611	4.50E-15	response to wounding
44283	7.24E-13	small molecule biosynthetic process
6519	4.25E-12	cellular amino acid and derivative metabolic process
44281	8.83E-12	small molecule metabolic process
42180	3.20E-11	cellular ketone metabolic process
**Group-IV (131)**		
9611	1.48E-16	response to wounding
6519	2.50E-15	cellular amino acid and derivative metabolic process
44283	3.85E-15	small molecule biosynthetic process
6575	5.25E-13	cellular amino acid derivative metabolic process
6725	3.12E-11	cellular aromatic compound metabolic process

B

GO-ID	p-value	Description
**Downregulated genes**		
9657	1.98E-05	plastid organization
16070	4.62E-05	RNA metabolic process
34641	8.66E-05	cellular nitrogen compound metabolic process
9791	1.27E-04	post-embryonic development
6807	1.38E-04	nitrogen compound metabolic process
**Upregulated genes**		
50896	8.32E-07	response to stimulus
42221	1.45E-06	response to chemical stimulus
9611	4.09E-06	response to wounding
19438	5.39E-06	aromatic compound biosynthetic process
6575	6.12E-06	cellular amino acid derivative metabolic process

Table S7. Summary of the GO term enrichment analysis of (**A**) common DEGs in Topping and OS treatment (**B**) OS-treatment specific DEGs

A

GO-ID	p-value	Description
**Group-I (27)**		
15979	2.2464E-07	photosynthesis
10218	4.2979E-07	response to far red light
10114	5.8731E-07	response to red light
9637	1.4031E-06	response to blue light
19684	0.000046463	photosynthesis, light reaction
**Group-III (48)**		
9805	4.2348E-07	coumarin biosynthetic process
9804	4.5633E-07	coumarin metabolic process
19748	0.000034831	secondary metabolic process
9699	0.000040738	phenylpropanoid biosynthetic process
9611	0.000044237	response to wounding
**Group-IV (141)**		
9611	1.628E-23	response to wounding
6519	2.7251E-21	cellular amino acid and derivative metabolic process
44283	5.135E-20	small molecule biosynthetic process
44281	6.7241E-19	small molecule metabolic process
6575	1.2598E-15	cellular amino acid derivative metabolic process

B

GO-ID	p-value	Description
**Downregulated genes**		
15979	1.14E-06	photosynthesis
9657	1.98E-05	plastid organization
42793	2.77E-05	transcription from plastid promoter
9658	5.15E-05	chloroplast organization
19684	0.000128	photosynthesis, light reaction
**Upregulated genes**		
9415	6.08E-06	response to water
6090	9.74E-05	pyruvate metabolic process
23	0.00012	maltose metabolic process
9414	0.000132	response to water deprivation
9628	0.000135	response to abiotic stimulus
